# Supplementary material for: New records of theropods from the latest Cretaceous of New Jersey and the Maastrichtian Appalachian fauna
Source: R Soc Open Sci. 2019 Nov 13;6(11):191206. doi: 10.1098/rsos.191206 (PMC6894593; doi:10.1098/rsos.191206)
Supplement: Supplementary Information [file rsos191206supp1.docx]

Supplementary Information for “New records of theropods from New Jersey inform faunal interchange in Maastrichtian North America”

Chase Doran Brownstein

Research Associate, Dept. of Collections and Exhibitions, Stamford Museum and Nature Center, Stamford, CT, **[chasethedinosaur@gmail.com](mailto:chasethedinosaur@gmail.com)**

This file includes:

1. Note on primary datasets.
2. Summary and loadings for the PCA of the Mt. Laurel tyrannosauroid tooth in a large matrix of theropod dentition.
3. Summary and loadings for the PCA of the Mt. Laurel dromaeosaurid tooth in a large matrix of theropod dentition.
4. Summary and loadings for the PCA of the Mt. Laurel dromaeosaurid tooth in a large matrix of North American paravian teeth.
5. Loadings and confusion matrix for the discriminant analysis of the Mt. Laurel dromaeosaurid tooth in a large matrix of North American paravian teeth.

Note on primary dataset number 1.

The modified dataset of Smith et al. (2005) (after Brownstein (2018a)) presented includes only the data for the Mt. Laurel dromaeosaurid tooth only. The analysis of the modified Smith et al. (2005) dataset with the Mt. Laurel tyrannosauroid tooth included used the measurements presented in Table 1 of the main text. Both primary dataset files are in .dat format, allowing them to be inserted into P.A.S.T by easily dragging them into the P.A.S.T interface.

Table 1. Loadings for the discriminant analysis of the Mt. Laurel dromaeosaurid tooth in a large matrix of theropod dentition.

|  | Axis 1 | Axis 2 | Axis 3 | Axis 4 | Axis 5 | Axis 6 | Axis 7 | Axis 8 | Axis 9 | Axis 10 | Axis 11 |
| --- | --- | --- | --- | --- | --- | --- | --- | --- | --- | --- | --- |
| E | 1.9732 | 2.1737 | -1.3971 | -0.96261 | 0.20518 | 2.7156 | -1.1334 | -1.9195 | -0.15326 | 0.23916 | 0.88876 |
| F | 1.4298 | 1.5523 | -1.153 | -0.56975 | 0.2744 | 2.0435 | -0.21883 | -2.3849 | -0.84038 | 0.15225 | 0.56897 |
| G | 3.8153 | 4.0056 | -2.6636 | -0.41396 | 0.19992 | 6.1943 | -3.5534 | -7.2749 | -1.3104 | 1.5251 | 2.4516 |
| H | 4.1232 | 4.3692 | -2.8791 | -0.69008 | 0.46843 | 6.4525 | -3.6736 | -7.132 | -1.8898 | 1.8462 | 5.0156 |
| I | 0.011076 | 0.0032784 | -0.020174 | 0.02615 | 0.01695 | -0.00029476 | 0.02662 | -0.029342 | -0.015934 | -0.007411 | 0.00042376 |
| J | 0.0030761 | 0.033027 | -0.016924 | 0.089193 | -0.021924 | 0.098936 | -0.055091 | -0.10818 | -0.010497 | 0.021251 | 0.055033 |
| K | 2.3717 | 1.1534 | 1.8955 | 0.81517 | -2.3595 | 1.0415 | -1.1566 | -0.46939 | 0.06961 | -0.52512 | 0.48912 |
| O | -0.12387 | -0.24959 | 0.55837 | -0.068578 | 0.56717 | 0.38893 | 0.38654 | 0.98605 | -1.4815 | 0.027467 | -0.50365 |
| Q | -0.68989 | 0.36734 | 0.40179 | -0.10668 | 0.44528 | -0.55632 | -1.1643 | 0.34864 | -0.62633 | 1.1396 | -0.45561 |
| R | -0.35675 | 0.016748 | 0.54158 | -0.27528 | 0.66573 | -0.44125 | -0.89322 | 0.18551 | -0.76833 | -2.0585 | 0.16919 |
| S | -0.47566 | -0.3232 | 1.6327 | -0.83131 | 1.6648 | 0.018355 | 0.48067 | -0.41637 | 0.56807 | 0.33408 | -0.030372 |
| T | -0.82618 | 0.52601 | 0.47978 | -0.10547 | 0.30618 | -0.68849 | 0.37635 | 0.2923 | -0.2489 | -0.029175 | -0.21358 |

Table 2. Confusion matrix for the discriminant analysis of the Mt. Laurel dromaeosaurid tooth in a large matrix of theropod dentition.

|  | North Carolina Tooth | Mt. Laurel | Gorgosaurus | Daspletosaurus | Tyrannosaurus | Troodon | Saurornithoides | Bambiraptor | Deinonychus |
| --- | --- | --- | --- | --- | --- | --- | --- | --- | --- |
| North Carolina Tooth | 1 | 0 | 0 | 0 | 0 | 0 | 0 | 0 | 0 |
| Mt. Laurel | 0 | 1 | 0 | 0 | 0 | 0 | 0 | 0 | 0 |
| Gorgosaurus | 0 | 0 | 10 | 3 | 1 | 0 | 0 | 0 | 0 |
| Daspletosaurus | 0 | 0 | 3 | 4 | 0 | 0 | 0 | 0 | 0 |
| Tyrannosaurus | 0 | 0 | 2 | 0 | 112 | 0 | 0 | 0 | 0 |
| Troodon | 0 | 0 | 0 | 0 | 0 | 6 | 0 | 0 | 0 |
| Saurornithoides | 0 | 0 | 0 | 0 | 0 | 0 | 8 | 0 | 0 |
| Bambiraptor | 0 | 0 | 0 | 0 | 0 | 0 | 0 | 9 | 0 |
| Deinonychus | 0 | 0 | 0 | 0 | 0 | 0 | 0 | 0 | 11 |

Table 3. Summary for the PCA of the Mt. Laurel dromaeosaurid tooth in a large matrix of North American paravian teeth.

| PC | Eigenvalue | % variance |
| --- | --- | --- |
| **1** | 15.7735 | 73.121 |
| **2** | 3.88624 | 18.015 |
| **3** | 1.33538 | 6.1904 |
| **4** | 0.4057 | 1.8807 |
| **5** | 0.170992 | 0.79267 |

Table 4. Loadings for the PCA of the Mt. Laurel dromaeosaurid tooth in a large matrix of North American paravian teeth.

|  | PC 1 | PC 2 | PC 3 | PC 4 | PC 5 |
| --- | --- | --- | --- | --- | --- |
| CBL | 0.36311 | 0.052943 | 0.022109 | 0.81836 | -0.44176 |
| CH | 0.81685 | 0.39839 | 0.1497 | -0.38935 | 0.0053977 |
| CBW | 0.18577 | -0.0090199 | 0.022263 | 0.4015 | 0.8965 |
| MDM | -0.27315 | 0.83228 | -0.47123 | 0.097825 | 0.032865 |
| DDM | -0.30295 | 0.38172 | 0.86865 | 0.089049 | 0.0051655 |

Table 5. Loadings for the discriminant analysis of the Mt. Laurel dromaeosaurid tooth in a large matrix of North American paravian teeth.

|  | Axis 1 | Axis 2 | Axis 3 | Axis 4 | Axis 5 |
| --- | --- | --- | --- | --- | --- |
| CBL | -0.33692 | 0.84118 | -0.010799 | 0.78881 | 0.085099 |
| CH | -0.55682 | 2.0018 | 0.60065 | 1.1881 | -0.98783 |
| CH | -0.20735 | 0.56001 | -0.12018 | 0.00987 | 0.021182 |
| MDM | 0.65866 | -0.061665 | 0.99995 | -0.41246 | 0.80876 |
| DDM | 0.7894 | -0.19168 | -0.32626 | -0.17373 | 0.041271 |

Table 6. Confusion matrix for the discriminant analysis of the Mt. Laurel dromaeosaurid tooth in a large matrix of North American paravian teeth.

|  | Mt. Laurel | Tar Heel |  | s | a | d | z | t | Pectinodon bakkeri | Aquilan cf. Richardoestesia gilmorei | Total |
| --- | --- | --- | --- | --- | --- | --- | --- | --- | --- | --- | --- |
| Mt. Laurel | 1 | 0 | 0 | 0 | 0 | 0 | 0 | 0 | 0 | 0 | 1 |
| Tar Heel | 0 | 1 | 0 | 0 | 0 | 0 | 0 | 0 | 0 | 0 | 1 |
|  | 0 | 0 | 34 | 0 | 0 | 0 | 0 | 0 | 0 | 0 | 34 |
| s | 10 | 1 | 90 | 183 | 18 | 16 | 66 | 1 | 8 | 37 | 430 |
| a | 1 | 0 | 2 | 1 | 15 | 4 | 4 | 2 | 2 | 0 | 31 |
| d | 2 | 3 | 11 | 5 | 13 | 80 | 12 | 3 | 2 | 0 | 131 |
| z | 0 | 0 | 9 | 0 | 2 | 3 | 20 | 0 | 1 | 0 | 35 |
| t | 0 | 0 | 0 | 0 | 2 | 0 | 0 | 66 | 14 | 0 | 82 |
| Pectinodon bakkeri | 0 | 0 | 2 | 0 | 0 | 0 | 0 | 0 | 43 | 0 | 45 |
| Aquilan cf. Richardoestesia gilmorei | 0 | 0 | 7 | 23 | 0 | 0 | 0 | 0 | 0 | 125 | 155 |
| Total | 14 | 5 | 155 | 212 | 50 | 103 | 102 | 72 | 70 | 162 | 945 |

Phylogenetic matrix

xread 141 65

Eoraptor

010100000000?000110000?02???????0100[01][12]100[01]

[01]10000101012[12]1100000[12]00?[01]10?[13]?00000000?012

210[01]00000001--001000000-0-0---0000

???????????????????????

Herrerasaurus

0100000000000000110000002000100010-10[23]10000-0--0---01-0-?000010-

1[012][01][01]000[02]0000000?000221000[01]100000-???0?

10000-200---0000???????????????????????

Eodromaeus

01???????????000410?000?30??10000?????????????????????????????0-

0[01]2000110[01]0000010000[01]1[01]0000[01]000020000?

00000-100---0010

???????????????????????

Coelophysis

010?000000000011010110000010?10010-00[012]10000-0--0---01-0-0030000-

[01][01][01]000[13]00000000[01]0??[01]

[01]100[01]0100000-000000000[03]00-0---001000011--1---0------

?????

Dilophosaurus

01[01]10020010100214111100120[01]11110?0-10[01]00001101???

01110000000000-

111000[34]1[01]00000010002310000[01]100000?0?20?0000-0-0--00?

0??????????????????00100

Ceratosaurus

00111-0000000000[34]1110001[23]001100010-10[23]

[12]000[01]1001100[23][23]10000010000-

[12][01]1[012]01[34]?

[01]000000100133100[01]011001[01]0[12]0[02]00100[02]0-

2010[01]0001022021000110202000000100

Genyodectes

0111110000000000??11000?30011000?0-20[012]000011001100221000?

000000-

1[01]1[01]01[34]2[01]00000000[01]13310[01]0010001[01]-2020010[01]00-2

10---0010

???????????????????????

Berberosaurus

??????????????????????????????????????????????????????????????0

-1010012310000000?01331[01][01]

???1001?-

1020?10000-200---0010?

??????????????????????

Noasaurus

????????????????4101000???????????????????????????????????????0-

01[01]100010000000100012100000000000?2?2?00000-0-0---0000

???????????????????????

Masiakasaurus

0?11???1?0???????10?000?30000001?210[12]

[01]10221[01]01011100100[02]0010000-

[01][01][12]0000100000001001[12]210[012]

[01]000000[01]00002000000-200---0010

??????????????????00110

Kryptops

??????????????????0?

001???????????????????????????????????????0-

1?1100010000000100?231[01]2[01]0000001?0?000022?0-0-0---0000

??????????????????0????

Rugops

0100000?1?001000?111001?????????????0???2?11??

00??????????????0-

1[01]?[12]000100000001000[23]310200000001?0?000000?

0-0-0---0000

???????????????????????

Abelisaurus

01?000???????000????00???????????

0-103[12][12]221100

???12210[12]00200000-

1[01][01]1000100000001?00331[01]

[12]0010011100000000100-1010100000???????????????????????

Aucasaurus

01?000??1?00?

00041010011??????????????????????????????????????0-

1[01][01]1000100000001000331[01][12][01]01[01]011100?

00000000-200---0000

??????????????????0?0??

Indosuchus

01000001100010004001001??0?10000?0-1[01]3[12][12][23]

[23]110[01]0011[23][23]1??0?000000-

?[01]?[12]0001000000010?0331??????????0???0??01

10-0-0---0000

???????????????????????

Majungasaurus

010000011[01]001000211100112001000010-103[12][12][23]

[23]110[01]0011221[12]200200000-

1[01][01]

[12]000100000001000[34]31[12]2000[01]000100000001220-20101000

00220210001101020000

?????

Skorpiovenator

01???????????000110?00?1?????????0-10222?[23]1100???1?11???0000000-

10[01][12]00[01]1000000010103310?0?11001[01]0?0?0?

00000-0-20200000???

????????????????????

Erectopus

????????????????????000???????????????????????????????????????0-

10[12]001210000000100033100[01]011010000000000100-1[01]0---0000

???????????????????????

Piatnitzkysaurus

????????????????1?00000??0???000??????????????????????????????0-

1110010100000000[01]10331000[02][01]10000000000001[12]0-

[12]1201000[01]0??????????????????00110

Eustreptospondylus

011110?0000?0000????000?30011000???1??10?0110?100022100?0100000-

11?0000100000000???[34]3100[01]0000000???00000??0-0-0-

--0010??

?????????????????????

Afrovenator

????????????????41010002??????????????????????????????????????

0-2010002100000000110431[01][01][01]2010?00-

0010001110-0-10000010?

?????????????????0?1?0

Dubreuillosaurus

0111100000000000410100023001100010-102100011001000221000?000000-

1[01]00000100000000100221[01][01][01]0000000-0010000110-0-0---0010?

?????????????????000?0

Duriavenator

0?11???0?0??00???101000??0010000?0-?02??1?110?100?[23]3100?0?00000-

1[01]100001?00000001??3310[01][01]00101[01]0-1010000110-[01]00---0010

??????????????????00?10

Megalosaurus

0???????????????41010002300100001?????????????????????????????0-

[12][01]100001000000001[01]0331[01][01][01][02]010100-1000001[12]

[12]0-2110[12][01]0010???????????????????????

Torvosaurus

01111001000100004101000230??10001??20???0011001000331???0?00000-

20200001000000001[01]0441[01][01][01]0110?

00-10100[01]1220-2[01]10100010??????????????????00210

Baryonyx

0[34]11101100110120?100102?

0011111010-11[12]1000110010020000000010000-

121000000000000[012]1[01]011[01]00[01]

[01]2111000202000000[12]00-10100021

??????????????????01011

Suchomimus

0411101100110120010010220011111?10-???

10001100100200000000[12]0000-

1[12][12]000000000000[012]1[01]0[12][12][01]00[01]

[01]1010002202000000[12]00-

10[12][01]0021???????????????????????

Irritator_Angaturama

0410001??0210121?????022?0??????1?????????????????????????????

0-12[12]0100000010101100-----------------------210-

101[01]0000???????????????????????

Spinosaurus

0312[01]0110121012141001123201111[12]110-?[01]??

1001010100[12]--------20000-[12][12][12]010000001010[01]100------

-----------------220-0---0021???????????????????????

Sinraptor

011110201002000030010002200?100010-1?[123]1[12]2311012011?21000??

00000-

[12]?1[01]00[01][23]100000000003310[01]00110?000000001?[02]?

0-210---0010

??????????????????00?00

Allosaurus

0211100110000000300100022000100010-1[01][12]00[23]

[23]11012111221[01][01]0000[01][02]00-

[12][012][12]0000200[01]0000[01]1[01]1[23][23]1[01]

[01]0011001100000011[12][12]0-

20201[01]000022021100111112000000000

Neovenator

02111120112000003100000??00110001?????????????????????????????

0-111001[12]20000000011[01]3[23]11100110010-

0000011110-2[01]20100010

??????????????????00211

Fukuiraptor

????????????????????000??0?1?0?0?

0-1[01]?0030110121?1[01]110[01]0??00000-

[01][01][012]00??1?000000[01]010[23][23]0[01][01]000[01]0?

0000000002220-[12]020?000

?????????????????????????

Australovenator

????????????????????????1001?000?

0-1[01][23]00?311012??1?[12]1?????00000-

1[01]1001[12]?000000000?[01]331?[01]?00[01]0??0-000001???0-??????

001????????????????????????

Acrocanthosaurus

0111110000000000310100022001100010-[12][01]

[12]100011001100[12]210000000000-

[12][01][012]0000?0000000[01]11[01]

[23]3[01]001[01]110111010[12]0010[01]00-

2[01]2[01]100010???????????????????????

Eocarcharia

????????????????310?000???????????????????????????????????????0-

101[03]00020000000011[01]321[01][01]0[02]

[12]10110-101[01]001110-100---0010

???????????????????????

Carcharodontosaurus

????????????????40010002?0?1?000??????????????????????????????

20201[13]000100000012110[34][34]1110010011[01]00

00[01]001[01][01]0-[01]021200010??????????????????00110

Giganotosaurus

01111101111?0000??0?000?2001?00010-

?1?1100110[01]1111221?00?10000202[01][12][03]00010000000[12]110[34]

[34]1[01][01]001[01]00000000001[12]220-

[12]0[12]0200010??????????????????00[12]10

Mapusaurus

????????????????4101000??0???000?0-

11[23]1000110[01]?1??221??000000020[12][01][01]

[013]00010000000[12]110[34][34]1[01][01]001100000000001[12]220-

0-[12][01]200010??????????????????[01]0[12]10

Proceratosaurus

01111100000000[01]0011100?2102110011100?

[23]103[03]110122[12]00010020000010-

1?[01]0000[13][01]0[01]000000[01]11[12]1000000000000002000[01]

[01]0-0-0---0010???

????????????????????

Eotyrannus

0121110000000000??10001??0210000?0-1211[01]4311013221[12]

[12]10000100000-

111[01]0001000000000[01]02[23]1000000000000002000110-0-0---0010

???????????????????????

Raptorex

0121110000000000400100022021100010-?2[01]104211003221??

10020000100-

?[01]100[01]0000[01]0000[01]0[01]1??100[01]

[02]1[01]000000000000100-100---0010?

?????????????????0?100

Alioramus

????????????????21110002102110001?????????????????????????????0-

?[01][01]00[01]0[13]10[01]0000[01]011??

1000011000100000000100-210---0010

??????????????????00100

Tyrannosaurus

0122110000000000411100023021000010-223[01]04[02]11003221[23]

[23]10000000[01]00-

[12][12][12][01]000000[01]0000[01]001[34][34]100001[01]0100020200122?

0-2[01]0---001022021101---0-0111200000

Compsognathus

01?????00????00?210?00?2100?100010-0?3[012]0?0

0-1-------------00000-0[01][12]00000?1--0[01]--2??-0--0-[01]-20--0--

00-10-000-0-0---0000

??????????????????10?00

Scipionyx

02?100-11122?001510000?23001100010-??2000?0-1-------------10000-??[012]

[01]0?0?11--01--[02]??-??-0-0-[02]0--0--?0-

10-000-0-0---0000???????????????????????

Ornitholestes

011100121021?0004101000230001000110[01]?

210300-1-------------00000-0110000211--0[01]--200-00-0-0-00--0---0-10-

000-0-0---0010

???????????????????????

Shuvuuia

????????????????000100?401000000?1100?2200

0-1-------------00?0210012000001--00----------------------------0-0-0---000-

??????????????????0?0??

Jianchangosaurus

1---------------0---00?20000-000?211021004110000001?22??000?

0011021[12]0?[03]0000000002003322200000011-2-

20010000-0-0---000-??

????????????????010?0

Erlikosaurus

1---------------0???00?30010?00110-002210011000000??2??

0000000110202000000000000200??2[02][02]00020011-2-

20010000-0-0---000-??

????????????????0?0??

Tsaagan

011100210021?000400?00?2[23]001000010-1?3[01]

[01]000-0--0---01-0-0000?00-1?1[01]0?0311--01--000-21-0-0-10--0--

00-00-000-0-0---0000

???????????????????????

Velociraptor

011000110?21?000[34]10?00?2[23]001100010-00310301101201?001?02?

030?00-

001[01]0?1?2[01]00000000012100000[01]0000?

0022000000-0-0---0[012]00100110-00000-----1

?????

Bambiraptor

01??00??????00004???00?230?10000??????????

0-0--?---??-?-??0???0-

0[01]1000[01]32[01]0000000001[12]10[01]0001000[01]??

002010000-0-0---0[02]1000000--1---12-----?????

Dromaeosaurus

0???000?10000000500100023001000010-1[01][12]

[01]033110121111111000100000-

[01]11000[01]3[12]010000[01]001[23]2100000[01]000100000000100-1

00---0000201110-000112----2?????

Saurornitholestes

01?????????????0????????20???000?0

-0221033[01]10121111[12]1[01][12]20100000-

[01]02[01]00[12]0[12][01][01]0000[01]000[12][12]

[12]22[12]-01000[01]00002010000-0-0---0000211110-00020-----100100

Buitreraptor

?????????????????????002?0??0000??????????????????????????????0-0[01]

[01]000[01]321--1----------------------------

-0-0-0-

--[13][12]00??????????????????1?1?0

Byronosaurus

0101000110?00000021100?30100?000?

1000211010-1-------------0000[12]00[12][12][01]0?[01][24]

[01]1--1---------------------

--------0-0-0---[13][12]0000000--1---22-----

?????

Zanabazar

0101000000000000[01]21100040100100011100311[35]?

0-1-------------0000[12]10[12][01][01]0?1[13]?[01]01000?000-32-2-

0-000-1--22-1[01]0000-000---2000?

??????????????????????

Troodon

01??????????????????000?010000001210121050[01][01]0000?

13322200000002101[01][01]00[134]1[01][01]0[01]0001000[

34][34]

[12]22[12]00[01]001100000010000-100---0000110110-00020-----100110

Richardoestesia_gilmorei

????????????????????????10????????????????????????????????????2?

001[01]0023[12]00[01]0000000[12][12]101000[02]00

0[01]0010[02]000000-0-0---000-200110-010112----0?

????

ML962

?????????????????????????????????0-

20100001100?0?0331?00?

10000??????????????????????????????????????????????????????????????????

?????????????

ML327

??????????????????????????????????????????????????????????????0-

1111000100100001?1134112[01]011001000000?00220-100---2010

???????????????????????

ML966

??????????????????????????????????????????????????????????????0-

11110001001000?1?1033111[01]01100100?000?10220-1010110010

???????????????????????

ML939

??????????????????????????????????????????????????????????????2

-001000[12]31

???0000000?01?[01]?2?100?0-

010?00?100-0-0---2000

????????????????????1??

Mt_Laurel_tooth

????????????????????

????????????????????

????????????????????

?????1?1000??00000??

?0??21?1??1101?0???2

001?2?0?202010001???

????????????????????

?
